# Molecular epidemiology and risk factors of *Anaplasma* spp., *Babesia* spp. and *Theileria* spp. infection in cattle in Chongqing, China

**DOI:** 10.1371/journal.pone.0215585

**Published:** 2019-07-15

**Authors:** Zuoyong Zhou, Kai Li, Yingying Sun, Junge Shi, Hexian Li, Yiwang Chen, Haoyue Yang, Xiao Li, Bi Wu, Xiaoxia Li, Zhiying Wang, Fangjun Cheng, Shijun Hu

**Affiliations:** 1 College of Animal Science, Rongchang Campus of Southwest University, Chongqing, China; 2 Veterinary Science Engineering Research Center of Chongqing, Chongqing, China; Institut national de la santé et de la recherche médicale - Institut Cochin, FRANCE

## Abstract

Tick-borne pathogens (TBPs) seriously affect cattle production and can be economically damaging. The epidemiology of these organisms in the Chongqing municipality of China is not well described. This study aimed to investigate the prevalence and risk factors of TBPs including *Anaplasma* spp., *Babesia* spp. and *Theileria* spp. in cattle in Chongqing municipality. The results showed that 43.48% (150/345) of cattle were infected with at least one TBP, of which single infections were detected in 104 (30.14%), double infections in 34 cattle (9.86%) and triple infections in 12 (3.48%) of the cattle. The overall prevalence of *Anaplasma* spp., *Theileria* spp. and *B*. *bigemina* were 22.32%, 23.19% and 7.24%, respectively. Among these, the prevalence of *A*. *bovis*, *A*. *central*, *A*. *phagocytophilum*, *A*. *platys*, *A*. *marginale*, *T*. *sinensisi* and *T*. *orientalis* were 8.41%, 7.83%, 4.93%, 4.35%, 2.61%, 22.32% and 2.60%, respectively. We could not detect *B*. *bovis*, *T*. *annulata*, *T*. *luwenshuni* or *T*. *uilenbergi* in cattle. Cattle ≥1-year-old were more likely to be infected with *Theileria* spp. [adjusted odd ratio (AOR) = 2.70, 95% CI = 1.12–6.56)] compared with younger cattle, while cattle ≥1-year-old had reduced susceptibility to *B*. *bigemina* (AOR = 0.14, 95% CI = 0.03–0.60). Cattle living at higher altitude (≥500 m) were more susceptible to *B*. *bigemina* (AOR = 6.97, 95% CI = 2.08–23.35) and *Theileria* spp. infection (AOR = 1.87, 95% CI = 1.06–3.32). The prevalence of *Theileria* spp. on farms with cats was significantly higher than that without cats (AOR = 2.56, 95% CI = 1.12–5.88). Infection with *A*. *bovis* and *A*. *central* were significantly associated with *A*. *phagocytophilum* infection. Furthermore, there were significant associations between *A*. *bovis* and *A*. *central* infection, *T*. *sinensisi* and *A*. *marginale* infection, and *B*. *bigemina* and *T*. *orientalis* infection. This study provides new data on the prevalence of *Anaplasma* spp., *Babesia* spp. and *Theileria* spp. in cattle in Chongqing, and for the first time we reveal a possible relationship between the afore-mentioned pathogens, which will help in formulating appropriate control strategies for these pathogens in this area.

## Introduction

Tick-borne pathogens (TBPs) have always attracted the attention of researchers, not only for their damaging influence upon livestock production but also for their public health threat [1]. Among the tick-borne diseases, anaplasmosis, babesiosis and theileriosis are the most important and are distributed widely. These organisms affect cattle worldwide [2]. Till now, five *Anaplasma* pathogens (*A*. *marginale*, *A*. *bovis*, *A*. *centrale*, *A*. *phagocytophilum*, and *A*. *platys*) have been reported to cause bovine anaplasmosis, of which *A*. *phagocytophilum* has been shown to infect a variety of animals and humans [3–5]. Two mainly *Babesia* pathogens, *Babesia bovis* and *B*. *bigemina*, were found responsible for bovine babesiosis [6], and three species of *Theileria* including *T*. *annulata*, *T*. *sinensis*, and *T*. *orientalis* (also named *T*. *sergenti*) were the main causative agents of bovine theileriosis [7–10], and recently, *T*. *luwenshuni* has also been detected in blood samples from cattle and yaks [9].

Numerous studies have reported the infection and prevalence of *Anaplasma* spp., *Babesia* spp. and *Theileria* spp. in cattle across many countries [4,5,11–16]. In China, there have also been many studies [9,17–22]. However, these studies usually focus on single pathogen infections, and records on pathogen co-infections, the risk factors, and the mutual influence of each pathogen in cattle are absent. In addition, studies relating to the aforementioned pathogens in cattle in China have mainly been restricted to the northwest region, while the information is very limited for southwest China.

The total number of cattle approximated 300 million at the end of 2015 in Chongqing, and is one of the economic pillars of animal husbandry in this city. However, the prevalence of *Anaplasma* spp., *Babesia* spp., and *Theileria* spp. in cattle in this area is unclear. The objectives of this study were 1) to detect *Anaplasma* spp., *Babesia* spp., and *Theileria* spp. in cattle in Chongqing, 2) to analyze the risk factors for infection of *Anaplasma* spp., *Babesia* spp., and *Theileria* spp., and 3) to evaluate the associations of the aforementioned pathogens in cattle in Chongqing.

## Materials and methods

### Study area

Chongqing municipality is located in the southwest of China, between the northern latitudes of 28.10°–32.13°, and eastern longitudes of 105.11°–110.11°. Its altitude ranges between 73.1 m at the Yangtze River in Wushan and 2796.8 m at Liangshan peak in Wuxi. The climate tends to be subtropical, with a monsoon/humid climate and has an average annual temperature of 16–18°C.

### Blood sample collection and DNA extraction

Three hundred and forty five sodium citrate anticoagulated blood samples were collected from 10 ranches located in Tongnan, Rongchang, Jiangjing, Changshou, Liangping, Kaizhou, Yunyang, Wushan, Fuling, and Qianjiang, from May 2016 to April 2017. The ranches were selected based on the number of cattle (≥50) and convenience of sampling. The sampled animals were randomly selected from apparently healthy cattle, and the information including gender and age of cattle, as well as the altitude and the existence of cats in ranches were recorded. The blood samples were sent back to the laboratory within an ice box. Whole blood genome was extracted using a Wizard Genomic extraction kit (Promega, Madison, WI, USA) according to the manufacturer's instructions. This study was approved by the Ethics Committee of Southwest. Consent was obtained from cattle owners before the collection of blood samples from their cattle by an experienced, practicing veterinarian.

### PCR detection of *Anaplasma* spp., *Babesia* spp., and *Theileria* spp.

*Anaplasma* spp. (*A*. *bovis*, *A*. *central*, *A*. *marginale*, *A*. *phagocytophilum* and *A*. *platys*), *Babesia* spp.(*B*. *bovis* and *B*. *bigemina*) and *Theileria* spp. (*T*. *annulata*, *T*. *sinensis*, *T*. *orientalis*, *T*. *luwenshuni* and *T*. *uilenbergi*) infections were detected by PCR or nested PCR using the primers reported in previous studies [7,23–29], the detail of primers can be found in S1 Table. The primers were synthesized by Bioligo Biotechnology Co., Ltd (Shanghai, China). The PCRs were performed according to the amplification programs in Table 1, with a volume of 12.5 μL in the reaction system including: 6.25 μL Premix Taq (containing TaKaRa *Taq*, dNTP Mixture and *Taq* Buffer) (Takara Dalian, China), 0.5 μL of each forward and reverse primer (20 μmol/L), 1 μL whole blood genome and 4.25 μL ddH_2_O. The amplified PCR products were photographed after electrophoresis in 1% agarose gels. The PCR amplification product were randomly selected for sequencing to verify the reliability of test.

**Table 1:** Overall prevalence of *Anaplasma* spp., *B*. *bigemina*and *Theileria* spp. infection in cattle in Chongqing of southwest China.

Counties	Prevalence of *Anaplasma* spp.(%)						Prevalence of *Theileria* spp.(%)			*BB* (%)
	*Anaplasma*	*APH*	*AC*	*AB*	*AM*	*APL*	*Theileria*	*TS*	*TO*	
Qianjiang	13.33(4/30)	6.67(2/30)	10.00(3/30)	6.67(2/30)	0(0/30)	0(0/30)	6.67(2/30)	0(0/30)	6.67(2/30)	0.00(0/30)
Changshou	16.00(7/50)	0(0/50)	2.00(1/50)	2.00(1/50)	2.00(1/50)	14.00(7/50)	2.00(1/50)	2.00(1/50)	0(0/50)	8.00(4/50)
Fuling	21.43(6/28)	3.57(1/28)	7.14(2/28)	10.71(3/28)	0(0/28)	0(0/28)	25.00(7/28)	21.43(6/28)	3.57(1/28)	39.29(11/28)
Liangping	25.00(50/20)	0(0/20)	0 (0/20)	10.00(2/20)	0(0/20)	15.00(3/20)	5.00(1/20)	0(0/20)	5.00(1/20)	0(0/20)
Rongchang	28.26(13/46)	13.04(6/46)	10.86(5/46)	15.21(7/46)	0(0/46)	4.35(2/46)	23.91(11/46)	21.73(10/46)	4.35(2/46)	2.17(1/46)
Wushan	28.13(9/32)	0(0/32)	6.25(2/32)	0(0/32)	25.00(8/32)	0(0/32)	66.66(23/32)	66.66(23/32)	0(0/32)	0(0/32)
Yunyang	21.31(13/61)	4.92(3/61)	14.75(9/61)	14.75(0/61)	0(0/61)	3.28(2/61)	18.03(14/61)	21.31(13/61)	4.92(3/61)	11.48(7/61)
Kaizhou	8.57(3/35)	2.86(1/35)	5.71(2/35)	0(0/35)	0(0/35)	0(0//35)	22.86(8/35)	22.86(8/35)	0(0/35)	5.17(2/35)
Tongnan	30.30(10/33)	12.12(4/33)	0(0/33)	27.27(9/33)	0(0/33)	0(0/33)	30.30(10/33)	30.30(10/33)	0(0/33)	0(0/33)
Jiangjin	60.00(6/10)	0(0/10)	30.00(3/10)	50.00(5/10)	0(0/10)	10.00(1/10)	60.00(6/10)	60.00(6/10)	0(0/10)	0(0/10)
Total	22.32(77/345)	4.93(17/345)	7.83(27/345)	8.41(29/345)	2.61(9/345)	4.35(15/345)	23.19(83/345)	22.32(77/345)	2.60(9/345)	7.24(25/345)

Note: APH: *A*. *phagocytophilum*; AC: *A*. *central*; AB: *A*. *bovis*; AM: *A*. *marginale*; APL: *A*. *platys*; TS: *T*. *sinensisi*; TO: *T*. *orientalis*; BB: *B*. *bigemina*.

### Risk factor analysis

Multivariable logistic regression was performed in SPSS for Windows (18.0 version, SPSS Inc., Chicago, IL, USA) to analyze factors associated with aforementioned infections. Adjusted odd ratios (AOR) and 95% confidence intervals (CI) were calculated. A *p*-value of <0.05 was considered statistical significant.

## Results

### Prevalence of *Anaplasma* spp., *Babesia* spp., and *Theileria* spp. infection

**Fig 1:**
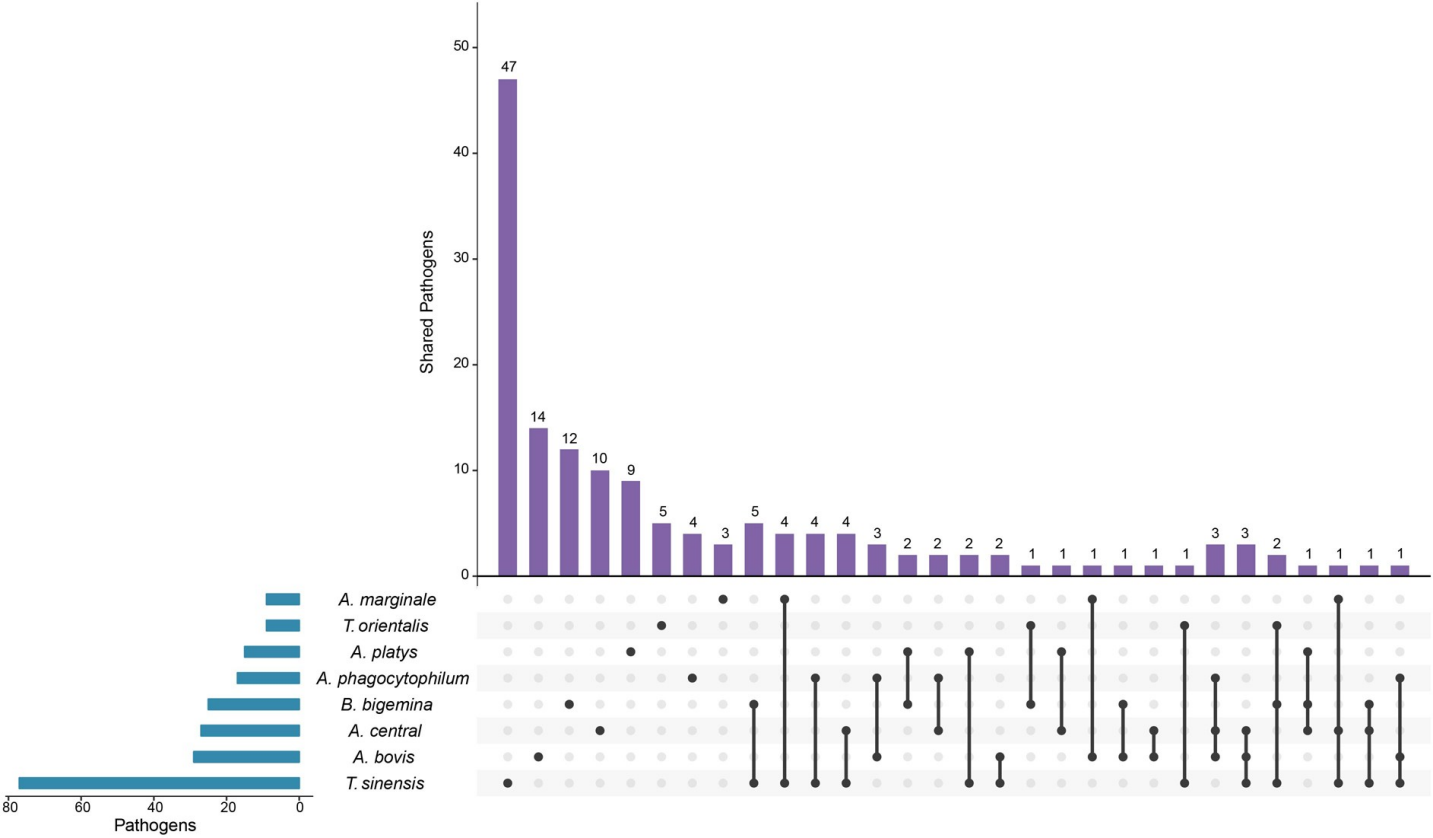
An UpSetR plot of *Anaplasma* spp., *Babesia* spp. and *Theileria* spp. infection in cattle from Chongqing of southwest China (n = 345). The blue horizontal coordinate columns represent the number of positive samples infected by pathogens. The purple vertical coordinate columns represent the number of positive samples infected by a single pathogen or multiple pathogens. The black dots represent the corresponding pathogens, and the dots connected by lines indicate co-infection of pathogens.

A total of 345 cattle in Chongqing were included in this study. Detailed information pertaining to infection is shown in Table 1 and Fig 1. The results showed that 43.48% (150/345) of cattle were infected with at least one TBP, of which single infections were detected in 104 (30.14%), double infections in 34 cattle (9.86%) and triple infections in 12 (3.48%) of the cattle. The overall prevalence of *Anaplasma* spp., *Theileria* spp., and *B*. *bigemina* in cattle were 22.32% (77/345), 23.19% (83/345), and 7.24% (25/345), respectively. Among the *Anaplasma* spp. detected, *A*. *bovis* (29/345, 8.41%) was the most prevalent species recorded, followed by *A*. *central* (27/345, 7.83%), *A*. *phagocytophilum* (17/345, 4.93%), and *A*. *platys* (15/345, 4.35%), while infection with *A*. *marginale* (9/345, 2.61%) was the lowest. Among the *Theileria* spp., *T*. *sinensisi* and *T*. *orientalis* infections in cattle were 22.32% (77/345) and 2.60% (9/345), respectively. In addition, we could not detect *B*. *bovis*, *T*. *annulata*, *T*. *luwenshuni*, or *T*. *uilenbergi* in this study.

### Risk factor analysis based on blood sample data

The prevalence of *Anaplasma* spp. (25.13%) and *Theileria* spp. (27.27%) in male cattle was significantly higher than that in females (*Anaplasma* spp.: AOR = 2.18, 95% CI = 1.05–4.52; *Theileria* spp.: AOR = 3.27, 95% CI = 1.47–7.25). Cattle ≥ 1-year were more likely to be infected with *Theileria* spp. than cattle aged below 1-year of age (25.00% vs. 20.99%), and the difference was statistically significant (AOR = 2.70, 95% CI = 1.12–6.56). In contrast, cattle ≥1-year had a lower risk of *B*. *bigemina* infection (AOR = 0.14, 95% CI = 0.03–0.60). Ranches at an altitude ≥500 m was found to be a risk factor for *B*. *bigemina* (AOR = 6.97, 95% CI = 2.08–23.35) and *Theileria* spp. infection (AOR = 1.87, 95% CI = 1.06–3.32). With exception of *Theileria* spp. (AOR = 2.56, 95% CI = 1.12–5.88), there were no significant associations between presence of cats and infection with *Anaplasma* spp., or *B*. *bigemina*, (Table 2).

**Table 2:** Multivariate analysis of selected factors and their association with *Anaplasma* spp., *B*. *bigemina*and *Theileria* spp. infection in cattle in Chongqing of southwest China.

Factors	Positive/Examined	Prevalence (%)	AOR (95% CI)	*p*-value
***Anaplasma* spp.**				
Gender				
Male	47/187	25.13	2.18(1.05–4.52)	0.037
Female	30/158	18.99	Reference	
Age				
≥1 year	58/264	21.97	1.68(0.75- 3.75)	0.204
<1 year	19/81	23.46	Reference	
Altitude				
≥500 m	31/156	19.87	0.62(0.34–1.10)	0.104
<500 m	46/189	24.34	Reference	
Cats presence				
Yes	46/204	22.55	1.87(0.89- 3.93)	0.100
No	31/141	21.99	Reference	
***B*. *bigemina***				
Gender				
Male	14/187	7.49	0.450(0.10–1.92)	0.280
Female	11/158	6.69	Reference	
Age				
≥1 year	13/264	4.92	0.14(0.03–0.60)	0.009
<1 year	12/81	14.81	Reference	
Altitude				
≥500 m	20/156	12.82	6.97(2.08–23.35)	0.002
<500 m	5/189	2.65	Reference	
Cats presence				
Yes	22/204	10.78	1.40(0.28–7.03)	0.681
No	3/141	2.13	Reference	
***Theileria* spp.**				
Gender				
Male	51/187	27.27	3.27(1.47–7.25)	0.004
Female	32/158	21.52	Reference	
Age				
≥1 year	66/264	25.00	2.70(1.12–6.56)	0.027
<1 year	17/81	20.99	Reference	
Altitude				
≥500 m	52/156	33.33	1.87(1.06–3.32)	0.031
<500 m	31/189	16.40	Reference	
Cats presence				
Yes	55/204	26.96	2.56(1.12–5.88)	0.025
No	28/141	19.86	Reference	

Note: AOR = adjusted odds ratio.

### Risk factor analysis based on pathogen co-infection

In order to evaluate the effect of specific pathogen infections and how they influence other pathogen infections within the same host, we considered each tested pathogen species as a potential risk factor in the analysis. The results of correlation analyses between each species of pathogen in infected cattle in Chongqing, are shown in Table 3. Infection with *A*. *bovis* and *A*. *central* were significantly associated with *A*. *phagocytophilum* infection, and *A*. *phagocytophilum* was more likely to increase the risk of *A*. *central* infection (AOR = 3.80, 95% CI = 1.10–13.18, *p* = 0.035). However, *A*. *central* was less likely to impact upon infection with *A*. *phagocytophilum* (AOR = 3.50, 95% CI = 0.97–12.59, *p* = 0.055). Furthermore, there was a significant association between *A*. *bovis* and *A*. *central* infection, *T*. *sinensisi* and *A*. *marginale* infection, and *B*. *bigemina* and *T*. *orientalis* infection (*p*<0.05). There were no significant associations between other pathogens that we aimed to identify.

**Table 3:** Correlation analysis based on species of *Anaplasma* spp., *Babesia* spp. and *Theileria* spp. infection in cattle in Chongqing, southwest China.

Targets	Factors	No. examed	No. positive (%)	AOR	95% CI	*p*-value
*APH*						
	*AB* infected	29	7 (24.14)	6.84	2.20–21.20	0.001
	Non-*AB* infected	316	10 (3.16)	Reference		
	*AC* infected	27	5 (18.52)	3.50	0.97–12.59	0.055
	Non-*AC* infected	318	12 (3.77)	Reference		
*AB*						
	*AC* infected	27	7 (25.93)	3.56	1.21–10.40	0.020
	Non-*AC* infected	318	22 (6.92)	Reference		
	*APH* infected	17	7(41.18)	7.09	2.29–21.97	0.001
	Non-*APH* infected	328	22(6.71)	Reference		
*AM*						
	*TS* infected	77	5 (6.49)	4.74	1.20–18.62	0.026
	Non- *TS* infected	268	4 (1.49)	Reference		
*BB*						
	*TO* infected	9	3 (33.33)	6.87	1.55- 30.47	0.011
	Non- *TO* infected	336	22 (6.55)	Reference		
*AC*						
	*AB* infected	29	7(24.14)	3.74	1.29–10.79	0.015
	Non- *AB* infected	316	20(5.33)	Reference		
	*APH* infected	17	5(29.41)	3.80	1.10- 13.18	0.035
	Non- *APH* infected	328	22(6.71)	Reference		
*TO*						
	*BB* infected	25	3(12.00)	6.84	1.52–30.82	0.012
	Non- *BB* infected	320	6(1.88)	Reference		
*TS*						
	*AM* infected	9	5(55.56)	4.77	1.21- 18.64	0.025
	Non- *AM* infected	336	72(21.43)	Reference		

Note: AOR: adjusted odds ratio; APH: *A*. *phagocytophilum*; AC: *A*. *central*; AB: *A*. *bovis*; AM: *A*. *marginale*; APL: *A*. *platys*; TS: *T*. *sinensisi*; TO: *T*. *orientalis*; BB: *B*. *bigemina*.

## Discussion

For the first time, this systematic study investigated the epidemiology of *Anaplasma* spp., *Babesia* spp. and *Theileria* spp. infection in cattle in Chongqing, China. The infection rate of *Anaplasma* spp. in our study was lower than that reported in Algeria [5] and in Tunisia [16], but higher than that reported in northwest China [30]. The prevalence of *A*. *bovis* (8.41%) in cattle in Chongqing was higher than that of cattle reported in other locations, where the prevalence varied from 3.9% to 6.2% [5,16,18,30]. In contrast, the prevalence of *A*. *centrale* (7.83%) was lower than that of cattle in previous studies (range between 12.1%-39.4%) [5,11,16,31,32]. Compared to the high prevalence of *A*. *marginale* in cattle in Madagascar (89.7%), north-eastern Uganda (82.9%) [11], South Africa (57%) [31], Thailand (39.1%) [13] and in China (31.6%)[33], we demonstrated a relatively low infection rate of *A*. *marginale* (2.61%) in Chongqing. In addition, 4.93% of cattle tested positive for *A*. *phagocytophilum* in this study, which was similar to the positivity rate (5.3%) of this pathogen in white yaks [30]. *A*. *platys* infection in cattle was first reported in Algeria [4], while Ben et al. reported a prevalence of *A*. *platy*s-like species (3.5%, 13/367) in cattle in Tunisia [34]. In this study, we noted a prevalence of *A*. *platys* (4.35%, 15/345) in cattle for the first time in Chongqing.

The prevalence of *B*. *bigemina* in this study was similar to previous research by Liu et al [21], and is lower than that reported in other provinces of China [19–21,35], South Africa [36], and in Tanzania [37]. However, the prevalence in our study was higher than that recorded in the Philippines [38]. In this survey, only *T*. *sinensisi* and *T*. *orientalis* were detected, with the prevalence being lower than *T*. *sinensisi* and *T*. *orientalis* infection rates recorded elsewhere [13,14,17]. Similar to the previous report [17], we did not detected *B*. *bovis* infection in cattle. The reason may be that 1) *B*. *bovis* infection in tick is usually lower than *B*. *bigemina*, which result a lower transmission rates of *B*. *bovis*, and 2) *B*. *bovis*-infected red blood cells usually accumulate in the capillary bed and leading to low parasitemia in circulating blood [17]. For the reasons that *T*. *annualata*, transmitted by *Hyalomma anatolicum anatolicum*, is mainly distributed in Northern China [39], *T*. *luwenshuni* and *T*. *uilenbergi*, both transmitted by *Haemaphysalis qinghaiensis* and *H*. *longicornis*, usually infected sheep and goats in China [27], and there is no evidence of above ticks existence in Chongqing. It was not strange that we did not detected *T*. *annulata*, *T*. *luwenshuni*, or *T*. *uilenbergi* infection in cattle from Chongqing.

There were 117 described species in the Chinese tick, 38 of which carry multiple pathogens [40], and most of the ticks including *H*. *anatolicum*, *H*. *qinghaiensis*, *H*. *longicornis*, *H*. *bispinosa*, *Rhipicephalus (Boophilus) microplus*, *R*. *sanguineus*, *Dermacentor abaensis*, *D*. *silvarum* and *D*. *nuttalli* were founded in northwest, northeast or central of China [39,41–44], and these ticks are responsible for transmission of a large amount of TBPs. However, the only reported tick specie in Chongqing was *R*. *microplus* [45], which was recorded to be the vector of *A*. *phagocytophilumin*, *A*. *marginale*, *B*. *bigemina* and *B*. *bovis* in China [40,46,47]. The differences in the prevalence of some parasites in this study compared to that reported previously in other studies in China or other countries, might be associated with geographical difference and variation in tick species.

Risk factor analysis revealed a significant correlation of altitude and age with the prevalence of *B*. *bigemina* and *Theileria* spp., which supported a previous report that there was a trend in increased seropositivity for *B*. *bigemina* infection with age [37]. In addition, gender is a risk factor associated with prevalence of *Anaplasma* spp. and *Theileria* spp., in cattle, which showed that male cattle had higher risk for these two type of pathogens infection, and the presence of cats in farm had positive effect on *Theileria* spp. infection in cattle from Chongqing, and the reasons for these phenomenon are not clear.

This study first took a single infection as a risk factor in evaluating the impact on infection with other pathogens. We found that cattle infected with *A*. *bovis* or *A*. *central* were more likely to be infected with *A*. *phagocytophilum*, and there was also a strong association between *A*. *bovis* and *A*. *central* infection. In addition, a very close relationship was observed for co-infection with *T*. *sinensisi* and *A*. *marginale*, and *B*. *bigemina* and *T*. *orientalis*. *Anaplasma* spp., *Babesia* spp., and *Theileria* spp. are all tick borne pathogens (TBPs), and some ticks can harbor mixed TBPs [40,47,48]. For the reasons that one species of TBP can be spread by different types of ticks, and equally that the same type of tick may also be the transmission vector for many species of TBPs, the significant correlation of the aforementioned pathogens might be attributed to the fact that infected cattle were bitten by ticks carrying different pathogens. From current data, it is not possible to estimate the chronological order of the aforementioned pathogen infections but there does appear to be significant relationships among some of these pathogens during infection of cattle. Parasite-parasite interaction may modify the impact of the pathogenic species and affect the performance and survival of host [49,50]. It is a pity that this study failed to evaluated the effect of above TBPs on health of cattle, since all the sampled animals in this study were apparently healthy, and we did not track the outcome of these cattle and the causes of their death. Further research should be conducted to elucidate the type of ticks present in Chongqing and the proportion of ticks that carry TBPs. Furthermore, attempts should be made to confirm whether a pathogen significantly increases the incident infection of other pathogens and the effects on production performance of cattle.

## Conclusions

The results of the present survey indicated that infection of cattle with *Anaplasma* spp., *Babesia* spp., and *Theileria* spp. is widespread in Chongqing. We provide a possible relationship between afore-mentioned pathogenic infections, which will help in formulating appropriate control strategies for these pathogens in this area.

## Supporting information

S1 Table Primers used for *Anaplasma* spp., *Babesia* spp. and *Theileria* spp. detection in cattle. (DOCX)

## Abbreviations

TBPs: tick-borne pathogens
AOR: adjusted odd ratio
95% CI: 95% confidence intervals (CI)
nPCR: nested PCR
ID: initial denaturation
FE: final extension
APH: *A*. *phagocytophilum*
AC: *A*. *central*
AB: *A*. *bovis*
AM: *A*. *marginale*
APL: *A*. *platys*
TS: *T*. *sinensisi*
TO: *T*. *orientalis*
BB: *B*. *bigemina*
