# Correction: Molecular epidemiology and risk factors of *Anaplasma* spp., *Babesia* spp. and *Theileria* spp. infection in cattle in Chongqing, China

**DOI:** 10.1371/journal.pone.0221359

**Published:** 2019-08-14

**Authors:** The PLOS ONE Staff

In the Abstract, there are errors in the fifth and sixth sentences. The correct sentences are: The overall prevalence of *Anaplasma* spp., *Theileria* spp. and *B*. *bigemina* were 22.32%, 24.06% and 7.25%, respectively. Among these, the prevalence of *A*. *bovis*, *A*. *central*, *A*. *phagocytophilum*, *A*. *platys*, *A*. *marginale*, *T*. *sinensisi* and *T*. *orientalis* were 8.41%, 7.83%, 4.93%, 4.35%, 2.61%, 22.32% and 2.61%, respectively.

In the Prevalence of *Anaplasma* spp., *Babesia* spp., and *Theileria* spp. Infection subsection of the Results, there are errors in the third sentence. The correct sentence is: The overall prevalence of *Anaplasma* spp., *Theileria* spp., and *B*. *bigemina* in cattle were 22.32% (77/345), 24.06% (83/345), and 7.25% (25/345), respectively. In the same paragraph, there are also errors in the fifth sentence. The correct sentence is: Among the *Theileria* spp., *T*. *sinensisi* and *T*. *orientalis* infections in cattle were 22.32% (77/345) and 2.61% (9/345), respectively.

There are errors in Tables 1 and 2. The publisher apologizes for the errors. Please see the correct Table 1 and Table 2 here.

**Table 1:** Overall prevalence of *Anaplasma* spp., *B*. *bigemina* and *Theileria* spp. infection in cattle in Chongqing of southwest China.

Counties	Prevalence of *Anaplasma* spp.(%)						Prevalence of *Theileria* spp.(%)			*BB* (%)
	*Anaplasma*	*APH*	*AC*	*AB*	*AM*	*APL*	*Theileria*	*TS*	*TO*	
Qianjiang	13.33(4/30)	6.67(2/30)	10.00 (3/30)	6.67(2/30)	0(0/30)	0(0/30)	6.67(2/30)	0(0/30)	6.67(2/30)	0.00(0/30)
Changshou	16.00(8/50)	0(0/50)	2.00(1/50)	2.00(1/50)	2.00(1/50)	14.00(7/50)	2.00(1/50)	2.00(1/50)	0(0/50)	8.00(4/50)
Fuling	21.43(6/28)	3.57(1/28)	7.14(2/28)	10.71(3/28)	0(0/28)	0(0/28)	25.00(7/28)	21.43(6/28)	3.57(1/28)	39.29(11/28)
Liangping	25.00(5/20)	0(0/20)	0 (0/20)	10.00(2/20)	0(0/20)	15.00(3/20)	5.00(1/20)	0(0/20)	5.00(1/20)	0(0/20)
Rongchang	28.26(13/46)	13.04(6/46)	10.87(5/46)	15.22(7/46)	0(0/46)	4.35(2/46)	23.91(11/46)	21.74(10/46)	4.35(2/46)	2.17(1/46)
Wushan	28.13(9/32)	0(0/32)	6.25(2/32)	0(0/32)	25.00(8/32)	0(0/32)	71.88(23/32)	71.88(23/32)	0(0/32)	0(0/32)
Yunyang	21.31(13/61)	4.92(3/61)	14.75(9/61)	0(0/61)	0(0/61)	3.28(2/61)	22.95 (14/61)	21.31(13/61)	4.92(3/61)	11.48(7/61)
Kaizhou	8.57(3/35)	2.86(1/35)	5.71(2/35)	0(0/35)	0(0/35)	0(0//35)	22.86(8/35)	22.86(8/35)	0(0/35)	5.17(2/35)
Tongnan	30.30(10/33)	12.12(4/33)	0(0/33)	27.27(9/33)	0(0/33)	0(0/33)	30.30(10/33)	30.30(10/33)	0(0/33)	0(0/33)
Jiangjin	60.00(6/10)	0(0/10)	30.00(3/10)	50.00(5/10)	0(0/10)	10.00(1/10)	60.00(6/10)	60.00(6/10)	0(0/10)	0(0/10)
Total	22.32(77/345)	4.93(17/345)	7.83(27/345)	8.41(29/345)	2.61(9/345)	4.35(15/345)	24.06(83/345)	22.32(77/345)	2.61(9/345)	7.25(25/345)

Note: APH: *A*. *phagocytophilum*; AC: *A*. *central*; AB: *A*. *bovis*; AM: *A*. *marginale*; APL: *A*. *platys*; TS: *T*. *sinensisi*; TO: *T*. *orientalis*; BB: *B*. *bigemina*

**Table 2:** Multivariate analysis of selected factors and their association with *Anaplasma* spp., *B*. *bigemina* and *Theileria* spp. infection in cattle in Chongqing of southwest China.

Factors	Positive/Examined	Prevalence (%)	AOR (95% CI)	*p*-value
***Anaplasma* spp.**				
Gender				
Male	47/187	25.13	2.18(1.05–4.52)	0.037
Female	30/158	18.99	Reference	
Age				
≥1 year	58/264	21.97	1.68(0.75–3.75)	0.204
<1 year	19/81	23.46	Reference	
Altitude				
≥500 m	31/156	19.87	0.62(0.34–1.10)	0.104
<500 m	46/189	24.34	Reference	
Cats presence				
Yes	46/204	22.55	1.87(0.89–3.93)	0.100
No	31/141	21.99	Reference	
***B*. *bigemina***				
Gender				
Male	14/187	7.49	0.45(0.10–1.92)	0.280
Female	11/158	6.96	Reference	
Age				
≥1 year	13/264	4.92	0.14 (0.03–0.60)	0.009
<1 year	12/81	14.81	Reference	
Altitude				
≥500 m	20/156	12.82	6.97 (2.08–23.35)	0.002
<500 m	5/189	2.65	Reference	
Cats presence				
Yes	22/204	10.78	1.40 (0.28–7.03)	0.681
No	3/141	2.13	Reference	
***Theileria* spp.**				
Gender				
Male	51/187	27.27	3.27 (1.47–7.25)	0.004
Female	32/158	20.25	Reference	
Age				
≥1 year	66/264	25.00	2.70 (1.12–6.56)	0.027
<1 year	17/81	20.99	Reference	
Altitude				
≥500 m	52/156	33.33	1.87 (1.06–3.32)	0.031
<500 m	31/189	16.40	Reference	
Cats presence				
Yes	55/204	26.96	2.56 (1.12–5.88)	0.025
No	28/141	19.86	Reference	

Note: AOR = adjusted odds ratio.
