# Supplementary material for: Molecular epidemiology and risk factors of Anaplasma spp., Babesia spp. and Theileria spp. infection in cattle in Chongqing, China
Source: PLoS One. 2019 Jul 15;14(7):e0215585. doi: 10.1371/journal.pone.0215585 (PMC6629066; doi:10.1371/journal.pone.0215585)
Supplement: S1 Table — (DOCX) [file pone.0215585.s001.docx]

**S1 Table . Primers used for *Anaplasma* spp*., Babesia* spp*.* and *Theileria* spp. detection in cattle**

| Pathogens | Methods | Primers | | Product (bp) | PCR program | | | | | | Reference |
| --- | --- | --- | --- | --- | --- | --- | --- | --- | --- | --- | --- |
|  |  |  |  |  | ID^b^ | Cycle | | | Cycles | FE^c^ |  |
| *A. bovis* | PCR | EE1  EE2 | 5'-TCCTGGCTCAGAACGAACGCTGGCGGC-3'  5'-AGTCACTGACCCAACCTTAAATGGCTG-3' | 1430 | 94℃  5min | 94℃  30s | 60℃  60s | 72℃  90s | 35 | 72℃  7min | [24] |
|  | nPCR^a^ | AB1f  AB1r | 5′-CTCGTAGCTTGCTATGAGAAC-3′  5′-TCTCCCGGACTCCAGTCTG-3′ | 551 | 94℃  5min | 94℃  30s | 55℃  60s | 72℃  90s | 35 | 72℃  10min | [26] |
| *A. phagocytephilum* | PCR | EE1  EE2 | 5'-TCCTGGCTCAGAACGAACGCTGGCGGC-3'  5'-AGTCACTGACCCAACCTTAAATGGCTG-3' | 1430 | 94℃  5min | 94℃  30s | 60℃  60s | 72℃  90s | 35 | 72℃  10min | [24] |
|  | nPCR | SP2f  SP2r | 5′-GCTGAATGTGGGGATAATTTAT-3′  5′-ATGGCTGCTTCCTTTCGGTTA-3′ | 641 | 94℃  5min | 94℃  30s | 55℃  60s | 72℃  90s | 35 | 72℃  10min | [26] |
| *A. centrale* | PCR | EE1  EE2 | 5'-TCCTGGCTCAGAACGAACGCTGGCGGC-3'  5'-AGTCACTGACCCAACCTTAAATGGCTG-3' | 1430 | 94℃  5min | 94℃  30s | 60℃  60s | 72℃  90s | 35 | 72℃  10min | [24] |
|  | nPCR | AC1f  AC1r | 5’-CTGCTTTTAATACTGCAGGACTA-3’  5’-ATGCAGCACCTGTGTGAGGT-3’ | 426 | 94℃  5min | 94℃  30s | 55℃  60s | 72℃  90s | 35 | 72℃  10min | [26] |
| *A. platys* | PCR | EE1  EE2 | 5'-TCCTGGCTCAGAACGAACGCTGGCGGC-3'  5'-AGTCACTGACCCAACCTTAAATGGCTG-3' | 1430 | 94℃  5min | 94℃  30s | 60℃  60s | 72℃  90s | 35 | 72℃  5min | [24] |
|  | nPCR | APf  APr | 5’-AAGTCGAACGGATTTTTGTC-3’  5’-CTTTAACTTACCGAACC-3’ | 506 | 94℃  5min | 94℃  30s | 55℃  60s | 72℃  90s | 35 | 72℃  5min | [25] |
| *A. marginale* | PCR | AMf  AMr | 5'-TGTCTAACCTTCTGCTGTTCGTTGC-3'  5'-ACACGAAACTGTACCACTGCCATGC-3' | 643 | 94℃  5min | 94℃  30s | 56℃  60s | 72℃  90s | 35 | 72℃  10min | [29] |
| *B. bovis* | PCR | B.Ba1f  B.Ba1r | 5’-AGTTGTTGGAGGAGGCTAAT-3’  5’-TCCTTCTCGGCGTCCTTTTC-3’ | 907 | 95℃  5min | 94℃  60s | 55℃  60s | 72℃  60s | 30 | 72℃  10min | [28] |
|  | nPCR | SBP4f  SBP4r | 5 '-GAAATCCCTGTTCCAGAG-3'  5' -TCGTTGATAACACTGCAA-3' | 503 | 95℃  5min | 94℃  60s | 55℃  60s | 72℃  60s | 30 | 72℃  10min | [28] |
| *B. bigemina* | PCR | B.N1f  B.N1r | 5’-GAGTCTGCCAAATCCTTAC-3’  5’-TCCTCTACAGCTGCTTCG-3’ | 879 | 95℃  5min | 94℃  60s | 55℃  60s | 72℃  60s | 30 | 72℃  10min | [28] |
|  | nPCR | RAP1af  RAP1ar | 5'-AGCTTGCTTTCACAACTCGCC-3'  5' -TTGGTGCTTTGACCGACGACAT-3 ' | 412 | 95℃  5min | 94℃  60s | 55℃  60s | 72℃  60s | 30 | 72℃  10min | [28] |
| *T. annulata* | PCR | TaN516f  TaN516r | 5'-GTAACCTTTAAAAACGT-3'  5'-GTTACGAACATGGGTTT-3' | 712 | 95℃  6 min | 94℃  60s | 55℃  60s | 72℃  60s | 30 | 72℃  10min | [23] |
| *T. orientalis* | PCR | Tserf  Tserr | 5'-CACGCTATGTTGTCCAAGAG-3’  5'-TGTGAGACTCAATGCGCCTA-3’ | 875 | 94℃  10min | 94℃  30s | 57℃  30s | 72℃  30s | 40 | 72℃  10min | [7] |
| *T. sinensis* | PCR | Tsinf  Tsinr | 5’-CACTGCTATGTTGTCCAAGAGATATT-3’  5’-AATGCGCCTAAAGATAGTAGAAAAC-3’ | 887 | 94℃  10min | 94℃  30s | 57℃  30s | 72℃  30s | 40 | 72℃  10min | [7] |
| *T. luwenshuni* | PCR | Tluw310  Tluw680 | 5’-GGTAGGGTATTGGCCTACTGA-3’  5’-TCATCCGGATAATACAAG-3’ | 340 | 94℃  3min | 94℃  30s | 57℃  60s | 72℃  60s | 40 | 72℃  7min | [27] |
| *T. uilenbergi* | PCR | Tuil310  Tuil680 | 5'-GGTAGGGTATTGGCCTACCGG-3'  5'-ACACTCGGAAAATGCAAGCA-3' | 340 | 94℃  3min | 94℃  30s | 57℃  60s | 72℃  60s | 40 | 72℃  10min | [27] |

a. nPCR: nested PCR.

b. ID: Initial denaturation

c. FE: Final extension
